# Age relationships with telomere length, body weight and body length in wild dugong (*Dugong dugon*)

**DOI:** 10.7717/peerj.10319

**Published:** 2020-11-11

**Authors:** Phaothep Cherdsukjai, Kittisak Buddhachat, Janine Brown, Manthanee Kaewkool, Anocha Poommouang, Patcharaporn Kaewmong, Kongkiat Kittiwattanawong, Korakot Nganvongpanit

**Affiliations:** 1 Phuket Marine Biological Center, Phuket, Thailand; 2 Department of Biology, Faculty of Science, Naresuan University, Phitsanulok, Thailand; 3 Excellence Center in Veterinary Bioscience, Chiang Mai University, Chiang Mai, Thailand; 4 Center for Species Survival, Smithsonian Conservation Biology Institute, Front Royal, VA, USA; 5 Department of Veterinary Biosciences and Public Health, Faculty of Veterinary Medicine, Chiang Mai University, Chiang Mai, Thailand

**Keywords:** Age, Growth, Telomere, Tusk, Senescence, Sirenia

## Abstract

The ability to estimate age and determine the growth status of free-ranging dugongs (*Dugong dugon*) is vital to providing insight into the basic biology of this endangered species. Currently, age estimation in dugong carcasses relies on counting dentin growth layer groups (GLGs) in tusks, but a disadvantage is they need to be intact. We explored whether measures of telomere length could be used as an alternative approach to age estimation in dugongs given that in other species, telomere length and age are inversely related. In this study, relative telomere length (rTL) was measured by qPCR in skin samples from 24 dugongs of varying ages determined by counts of GLGs. In addition, relationships between age by GLG counts and body weight and length and were examined. Our findings indicate that age estimated by GLGs was negatively correlated with telomere length using the logistic formula with a rate of telomere attrition of approximately 0.036 rTL/year between the ages of 5–20 years. By comparison, both body weight and length were positively correlated with GLG-based age, with growth rates of ~8.8 kg/year for weight and ~3.58 cm/year for length, respectively. After that, growth rates slowed substantially and then plateaued. The results suggest that physical maturity in dugongs occurs at 20 years of age and that measures of rTL might serve as a tool for age estimation in dugongs, living and deceased.

## Introduction

The dugong (*Dugong dugon*) is one of four species belonging to the Order Sirenia, Family Dugongidae. It is distinct compared to other marine mammals in being almost entirely herbivorous. In Thailand, dugongs are found along the Andaman Sea and Gulf of Thailand coastlines. Of the estimated 200–250 dugongs in Thailand, about 70% reside in the waters around the island of Koh Libong. Dugongs are listed as Vulnerable on the IUCN Red List (Marsh & Sobtzick, 2019), with threats primarily related to anthropogenic activities such as coastal development, habitat degradation, pollution, fishing nets, boat strikes and poaching. There are five laws in Thailand that can protect dugongs and seagrass (their main food source): (i) Fisheries Act, B.E. 2490 (1947); (ii) National Park Act, B.E. 2504 (1961); (iii) Export and Import of Goods Act, B.E. 2522 (1979); (iv) Wildlife Preservation and Protection Act, B.E. 2535 (1992); and (v) the Convention on International Trade in Endangered Species (CITES) (Adulyanukosol, Ellen & Potchana, 2010). All laws prohibit the killing, taking, possessing and trading of dugongs or their body parts in Thailand, while CITES bans these activities internationally. Still, dugong populations continue to decline (Marsh & Sobtzick, 2019), including in Thailand.

Dugongs have a life span of around 60–70 years (Marsh, 1995) and reach sexual maturity at ~10–12 years of age (Whittemore et al., 2019); however, age at physical maturity is unknown. Dugongs with unknown birth dates have been aged by analysis of dentition (Lanyon & Sanson, 2006). They have six pairs of cheekteeth that move forward with age. The dental formula for adult dugongs is 1/0, 0/0, 3/3, 3/3 (Marsh, 1980), which means they have one incisors, no canines, three premolars and three molars on each side of the upper jaw, and three premolars, and three molars on the lower jaw (Marsh, 1980; Myers, 2002). Premolars erupt at birth and fall out or are partially resorbed, while molars progressively erupt posteriorly during growth (Lanyon & Sanson, 2006; Mitchell, 1973). Two pairs of upper incisors are formed; the anterior pair is vestigial, nonfunctional and deciduous, while the posterior pair is permanent and forms the tusks. The tusks erupt later, primarily in males, but occasionally in females (Marsh, 1980). The tusks are the only teeth present throughout life, and so have been used for age estimation (Marsh, 1980; Mitchell, 1976; Read, Hohn & Lockyer, 2018) through counts of dentin growth layer groups (GLGs). The technique was first described by Scheffer in 1970 (Scheffer, 1970) and is now broadly accepted for age estimation of dugongs (Adulyanukosol, Amorena & Miyazaki, 1998; Kasuya & Nishiwaki, 1978; Marsh, 1980; Mitchell, 1976; Mitchell, 1978; Read, Hohn & Lockyer, 2018; Scheffer, 1970). There is a seasonal deposition of dentin, resulting in one GLG per year. The technique is applied widely to age estimates of other marine mammal species, including manatees (*Trichechus manatus*) (Marmontel et al., 1996), gray seals (*Halichoerus grypus*), walruses (*Odobenus rosmarus*), killer whales (*Orcinus orca*), California lion seals (*Zalophus californianus*) (Rust et al., 2019) and bottlenose dolphins (*Tursiops truncatus*) (Read, Hohn & Lockyer, 2018). Most species estimated for age by GLG in mineralized tissues (bones and teeth) were free-ranging animals with unknown age, contributing to trouble in how reliable this technique is. However, a few studies revealed the accuracy of age estimation by counting GLG in tooth dentin of sea lion as 71–79% (Rust et al., 2019) and in manatee bone as approximately *R*^2^ of 0.97 between known age and estimated age (Marmontel et al., 1996). Apart from teeth, there are other dense connective tissues that can be used for age estimation; for example, ear plugs in baleen whales, (Lockyer, 1984), periotic bones in manatees (Marmontel et al., 1996), and tympanic bullae in bowhead whales (*Balaena mysticetus*) (Sensor et al., 2018) and minke whales (*Balaenoptera acutorostrata*) (Sukhovskaya et al., 1985).

One problem with counts of GLGs for age estimation is that they are not accurate for animals with incomplete tusks (Adulyanukosol, Amorena & Miyazaki, 1998; Read, Hohn & Lockyer, 2018). For that reason, measurement of telomere length to estimate age may be an alternative approach. Telomeres are composed of repeating nucleotides at the ends of chromosomes and important for maintaining chromosome integrity and protecting against DNA deterioration or damage (Fathi et al., 2019; Shay & Wright, 2000). They shorten with each cell division throughout the life of an organism (Fathi et al., 2019; Hewakapuge et al., 2008; Vleck, Haussmann & Vleck, 2003), and so measures of telomere length have been used to estimate age in humans (*Homo sapiens*) (Hewakapuge et al., 2008; Kaewkool et al., 2020; Karlsson et al., 2008) and other mammalian species, including mice (*Mus musculus*) (Callicott & Womack, 2006; Whittemore et al., 2019), dogs (*Canis lupus familiaris*) (Adulyanukosol, Amorena & Miyazaki, 1998; Fick et al., 2012; McKevitt et al., 2002), cats (*Felis catus*) (Mitchell, 1976), goats (*Capra hircus*) (Whittemore et al., 2019), Asian elephants (*Elephas maximus*) (Buddhachat et al., 2017; Whittemore et al., 2019), Australian sea lions (*Neophoca cinerea*) (Izzo et al., 2011), humpback whales (*Megaptera novaeangliae*) (Olsen et al., 2014) and bottlenose dolphins (Whittemore et al., 2019). Most of these studies used peripheral blood to determine telomere length and found generally negative relationships with age (*R*^2^ < 0.3), with large variation potentially due to extrinsic (nutrition, behavior and environment) or intrinsic (rate of telomere shortening, initial telomere length, metabolic rate and genetics) factors contributing to low or no significant relationships between age and telomere length measurements using qPCR (Buddhachat et al., 2017; Fick et al., 2012; Hewakapuge et al., 2008; Izzo et al., 2011; Kaewkool et al., 2020; Karlsson et al., 2008). However, in humpback whales, the telomere length measurement was significantly correlated to age with an *R*^2^ = 0.244 (Olsen et al., 2014). Recently, Whittemore et al. (2019), measured telomere length in a variety of species and found there was no correlation between initial telomere length and lifespan, but rather it was related to telomere attrition rate. In contrast, a previous study of Fick et al. (2012) demonstrated that average telomere length correlated to average of lifespan across different breeds of dogs. This might indicate that the variation in telomere length affects lifespan only within species due to the same genetic background, but not for between species.

An advantage of telomere length measurement over the GLG technique is it can be done on living or deceased animals, does not require intact carcasses or tissues, and presumably can be applied to a variety of tissue types, such as skin, muscle and leukocytes (Daniali et al., 2013; Dlouha et al., 2014). The purpose of this study was to measure telomere length in dugong skin samples and determine how it correlates with chronological age estimated by GLG counts in tusks. We also examined relationships between age and body weight and length. Our hypothesis is that age estimates by GLG counts will correlate negatively with telomere length, and positively with body length and body weight. The goal is to find alternative approaches for age estimation of dugongs, particularly in those with incomplete tusks, to aid in population demographic analyses and studies of species biology.

## Materials and Methods

### Animals

**Figure 1:**
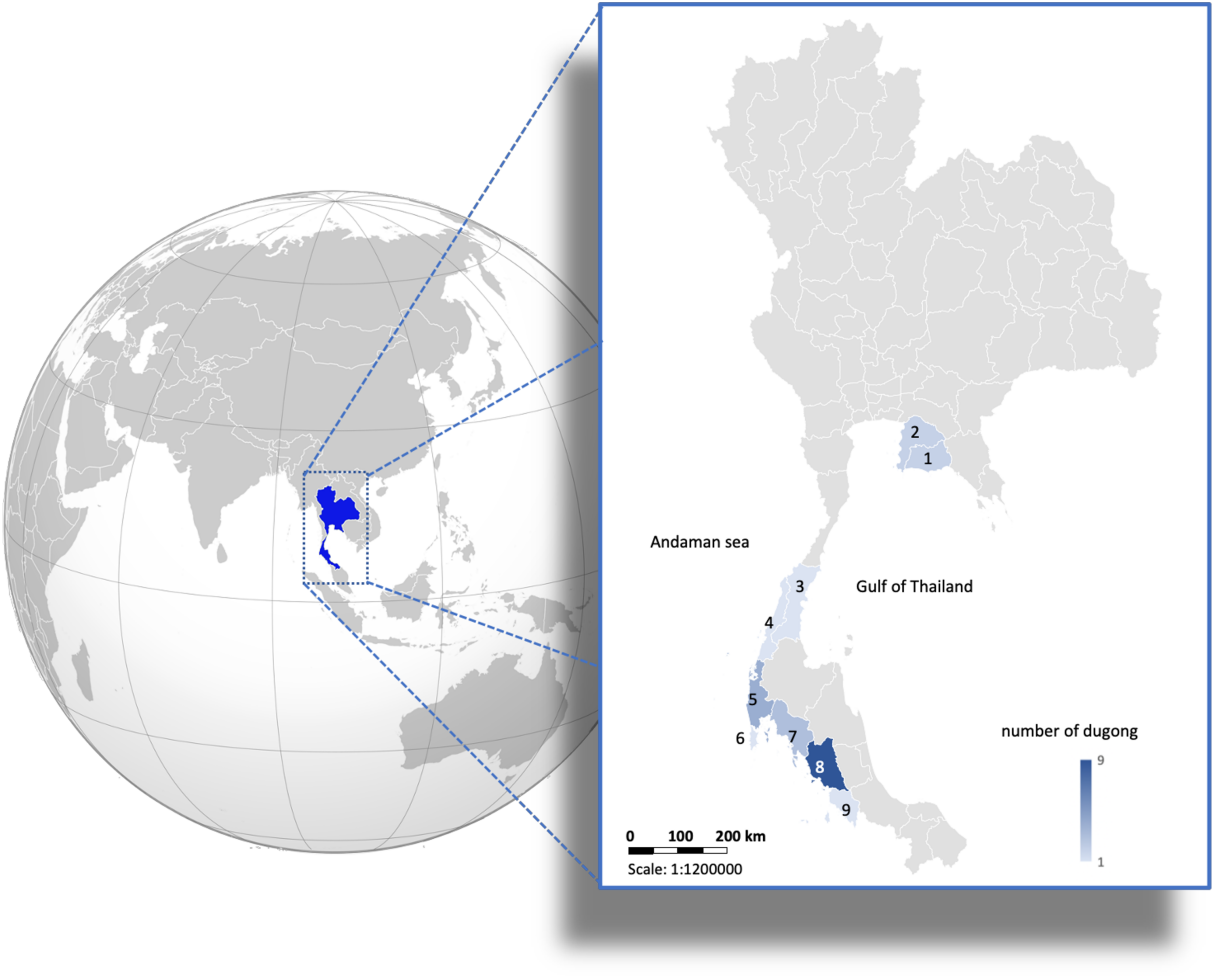
Distribution of 24 carcasses of stranded dugongs found along the coastlines of the Gulf of Thailand and Andaman sea among nine provinces. 1 = Rayong (*n* = 2); 2 = Chonburi (*n* = 2); 3 = Chumphon (*n* = 1); 4 = Ranong (*n* = 1); 5 = Phangnga (*n* = 4); 6 = Phuket (*n* = 1); 7 = Krabi (*n* = 3); 8 = Trang (*n* = 9); 9 = Satun (*n* = 1). (Photo created in Microsoft Excel, version 16.36, 20041300, licensed to Chiang Mai University).

Whole tusks from 24 stranded dugong (*Dugong dugon*) carcasses (female = 16, male = 8) found along the Gulf of Thailand and Andaman Sea coastlines of Thailand (Fig. 1) were obtained from the Phuket Marine Biological Center (Phuket, Thailand). Skin tissue samples (2 × 2 cm) from these carcasses were obtained and preserved in 95% ethanol for DNA extraction. According to the Animals for Scientific Purposes Act, B.E. 2558 (2015), since a part of this study was performed on carcasses of stranded dugongs, no ethical approval was required, as confirmed by the Animal Ethics Committee, Faculty of Veterinary Medicine, Chiang Mai University (License number U1006312558).

### Measures of dentin GLGs

**Figure 2:**
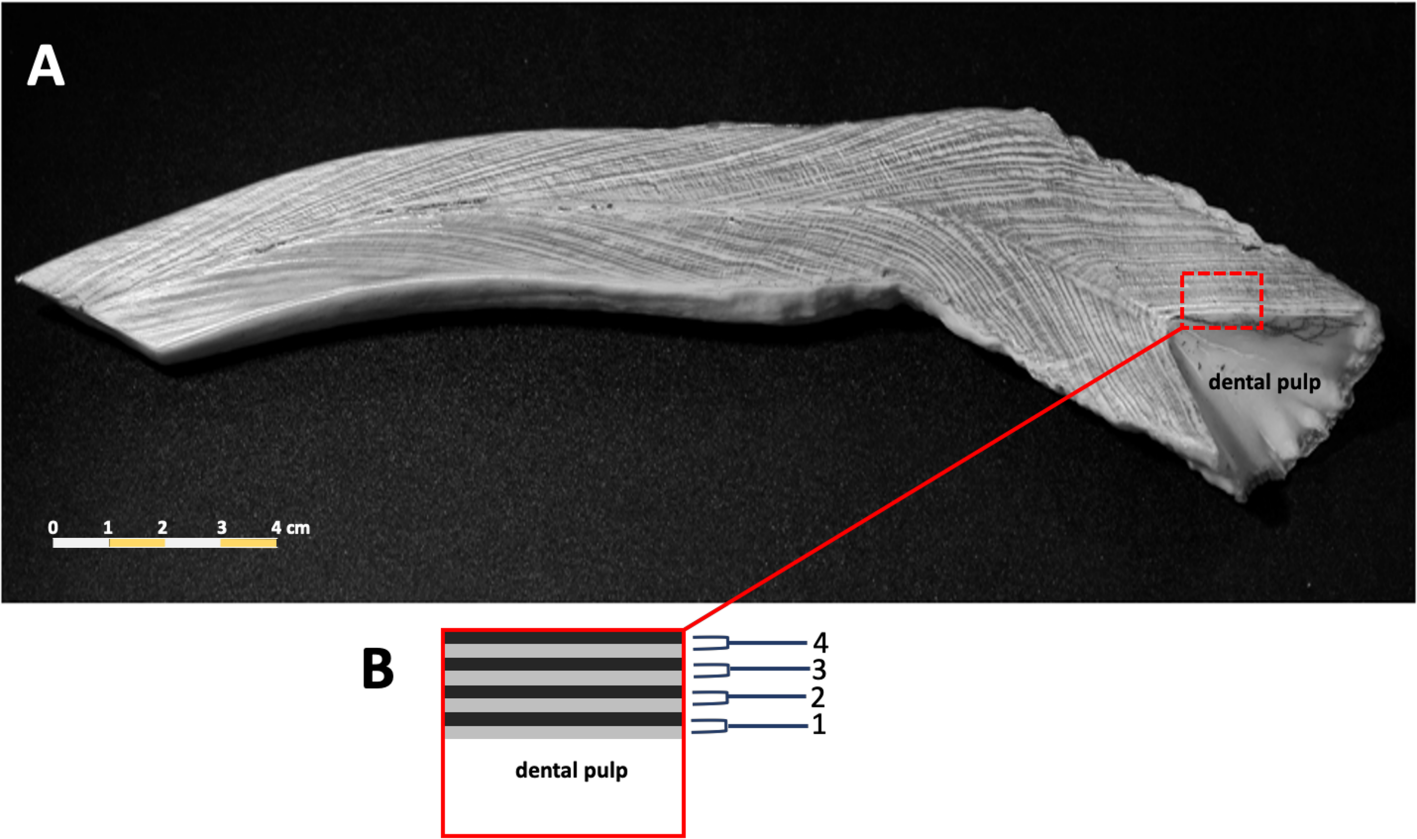
A representative photo of counted dentinal growth layer groups in the tusk of a 47+ year-old female dugong (A); each GLG was represented by one light and one dark band in the dental pulp from base to tip of the tusk (B).

Counts of dentin GLGs followed previous protocols (Adulyanukosol, Amorena & Miyazaki, 1998; Mitchell, 1978), incomplete or worn tusks were excluded from this study. The tusks were sectioned longitudinally in the mid-sagittal plane using a Buehler Isomet low speed saw (Struers Minitom, Denmark), polished using progressively finer grades of sandpaper (150–1,200), and then etched in 10% formic acid for 1–3 h. Tusks were washed in tap water for 10 min to remove the etching reagent, followed by immersion in 100% acetone for 2–3 min to increase the clarity of the GLGs. Samples were rewashed in tap water for 12 h to remove the formic acid. The etched tusk was dried at room temperature for 1–2 days and then rubbed with laser toner powder (Canon, Thailand). The number of GLGs in each tusk was counted in triplicate (Fig. 2).

### DNA extraction and real-time PCR

Skin samples were extracted using genomic DNA extraction kits according to manufacturer’s instructions (DNeasy Blood & Tissue Kit, QIAGEN, Germany) at the Faculty of Veterinary Medicine, Chiang Mai University. DNA was measured qualitatively and quantitatively by agarose gel electrophoresis and spectrophotometry at *A*_260_, respectively, with an *A*_260_ of 1 = 50 ng/μl. Subsequently, dugong DNA (50 ng) was used for measurement of telomere length by qPCR (Buddhachat et al., 2017; Cawthon, 2002; Kaewkool et al., 2020). A reaction consisted of 1X SensiFAST^™^ SYBR^®^ No-ROX Kit (Bioline, England) was used and contained telomere primer concentrations of 270 nM of tel 1 5′ GGTTTTTGAGGGTGAGGGTGAGGGTGAGGGTGAGGGT 3′, and 900 nM of tel 2 5′ TCCCGACTATCCCTATCCCTATCCCTATCTATCCCTA 3′ in a total volume of 10 μl. Additionally, the acidic ribosomal phosphoprotein 36B4 (36B4) was single copy gene used as a internal control to measure relative quantity of telomeric repeat. The sequence of primer of 36B4 was forward CAGAGTGAYGTGCAGCTGAT and reverse AAGCACTTCAGGGTTGTAGATGCTGCC and their concentration performed for DNA amplification was 400 nM of each primer.

The cycling profile for the telomere PCR was as follows: 40 cycles of 95 °C for 15 s and 65 °C for 2 min. For 36B4 as a single copy gene, 30 cycles of 95 °C for 15 s and 65.5 °C for 1 min were applied with an Eco Real-Time PCR System (Illumina, San Diego, CA, USA). After that, cycle threshold (*C*_t_) values were acquired from qPCR and used to calculate the relative telomere length (rTL) derived from the following formula: 2^−delta(ct(telomere)−ct(36B4 gene))^. Each individual was done in triplicate for measuring rTL. The rate of telomere loss was obtained from the slope of the linear regression model (rTL/year).

### Statistical analyses

A histogram for each parameter was created to determine the data distribution. To determine the variability of experimental error and biological error for rTL measurement using qPCR, coefficients of variation (CV; mean/standard deviation × 100) obtained from intra-individual (triplicate) and inter-individual within the same stage (premature and mature) presented the error caused by experiment and biological factor, respectively. Relationships among age, body weight, body length and rTL were determined by logistic, logarithm or linear regression models depending on the variable. A logistic model determined correlations between weight-age, length-age and rTL-age as either $Y = \; \textstyle{L \over {1 + b{e^{ - \left( {c + {\rm \beta} X} \right)}}}}$ with $b \ne 0,\; b > 0$ or $Y = \; \textstyle{L \over {1 - b{e^{ - \left( {c + {\rm \beta} X} \right)}}}}$ with $b \ne 0,\; b < 0\;$and *L* (limiting value) or the curve’s maximum value. Weight-rTL and length-rTL were fitted by logarithm function as $Y = {\rm \beta} \log \left( X \right) + c$, and weight-length was explained by a linear regression model as $Y = {\rm \beta} X + c$. The model for age estimation by rTL measurement was determined for accuracy by *R*^2^ and precision by residual stand error based on the correlation of age estimated by GLG counts and by rTL using a linear regression model. Further, we created age cutoffs to analyze growth stages of immature and mature dugongs by coefficients of variation (CV) using 10-year periods between 5 and 60 years of age. After obtaining an age cutoff, dugong samples were divided into two classes: <20 or ≥20 years. A linear regression model was used to determine growth rates in body weight and length, as well as the rate of telomere attrition. In plotting a graph, all data points including the replicating data of each individual of rTL were used for creating the visualized graphs of their relationship. Differences between body weight, length and rTL between immature and mature dugongs were determined by Student’s *t*-tests. Data for dugongs <20 years old were available for males and females; however, males >20 years old were unavailable.

## Results

### Age determination by GLG counts

Chronological ages estimated for 24 tusks by counts of GLGs ranged from 5 to 69 years (Table 1) and were used to determine correlations between age, rTL and body morphometrics.

**Table 1:** Stranding information for the 24 dugongs in this study, and age estimates by counted dentinal growth layer groups from intact tusks.

ID	Stranding information		Body information			Age* (year)
	Date (M/D/Y)	Location	Sex	Length (cm)	Body (kg)	
307	24 January 2010	Libong Island, Trang	Female	277	358	54
300	5 October 2009	Samran Beach, Trang	Female	224	235	11
299	28 September 2009	Libong Island, Trang	Female	281	335	63
292	2/2009	Ao Nang Beach, Krabi	Female	282	305	41
291	1 February 2009	Ko Sarai Island, Satun	Female	263	317	67
285	26 July 2008	Map Ta Phut, Rayong	Female	200	–	24
283	9 April 2005	Ko Lanta Yai Island, Krabi	Female	300	–	38
272	30 August 2007	Sattahip, Chonburi	Male	245	–	13
258	12 August 2006	Bang Sare, Chonburi	Female	231	200	12
243	26 December 2004	Ko Pa Yung Island, Phangnga	Male	275	310	23
236	1 June 2004	Ko Klang Island, Phangnga	Female	250	–	69
234	22 April 2004	Ko Yao Island, Phangnga	Female	256.5	297	27
143	28 November 2001	Libong Island, Trang	Female	199.5	142	5
129	6 December 2000	Khao Mai Kaeo, Trang	Female	225	258	43
098	14 October 1998	Kram, Rayong	Male	214	180	16
084	3 August 1998	Wichit, Phuket	Male	219	184	8
078	19 May 1998	Lamae, Chumphon	Female	231	151	34
058	6 January 1997	Libong Island, Trang	Male	250	245	15
057	2 January 1997	Libong Island, Trang	Female	258	281	14
048	3 March 1996	Suk Samran, Ranong	Female	271	293	43
047	25 January 1996	Ko Yao Island, Phangnga	Male	221	143	6
038	21 June 1995	Libong Island, Trang	Male	257	250	14
036	31 March 1995	Bo Hin, Trang	Female	273	272	34
016	11 May 1993	Muang Krabi, Krabi	Male	254	–	16

**Note:**

* Age estimated by counted dentinal growth layer groups in tusks.

### Determination of variability of rTL measurement

In the present study, we tested the variability of rTL measurement using real-time PCR and if it was caused by differences in experimental design or biological factors as shown in Table 2. Our results revealed lower CVs obtained from intra-individual samples across both age groups compared to inter-individual values. These results suggest that although the experiment error had a relatively large variability, the biological factor (ages) appeared to be the main factor accounting for the variability in rTL.

**Table 2:** Intra- and inter-individual variability of rTL measurements by coefficient of variation (%CV) for premature and mature dugongs using real-time PCR.

Stage	Intra-individual (%)	Inter-individual (%)
Premature (<20 years)	38.97	47.02
Mature (≥20 years)	37.44	91.65
Overall mean	38.20	69.34

### Correlation between age and GLG counts, rTL and body morphometrics

**Figure 3:**
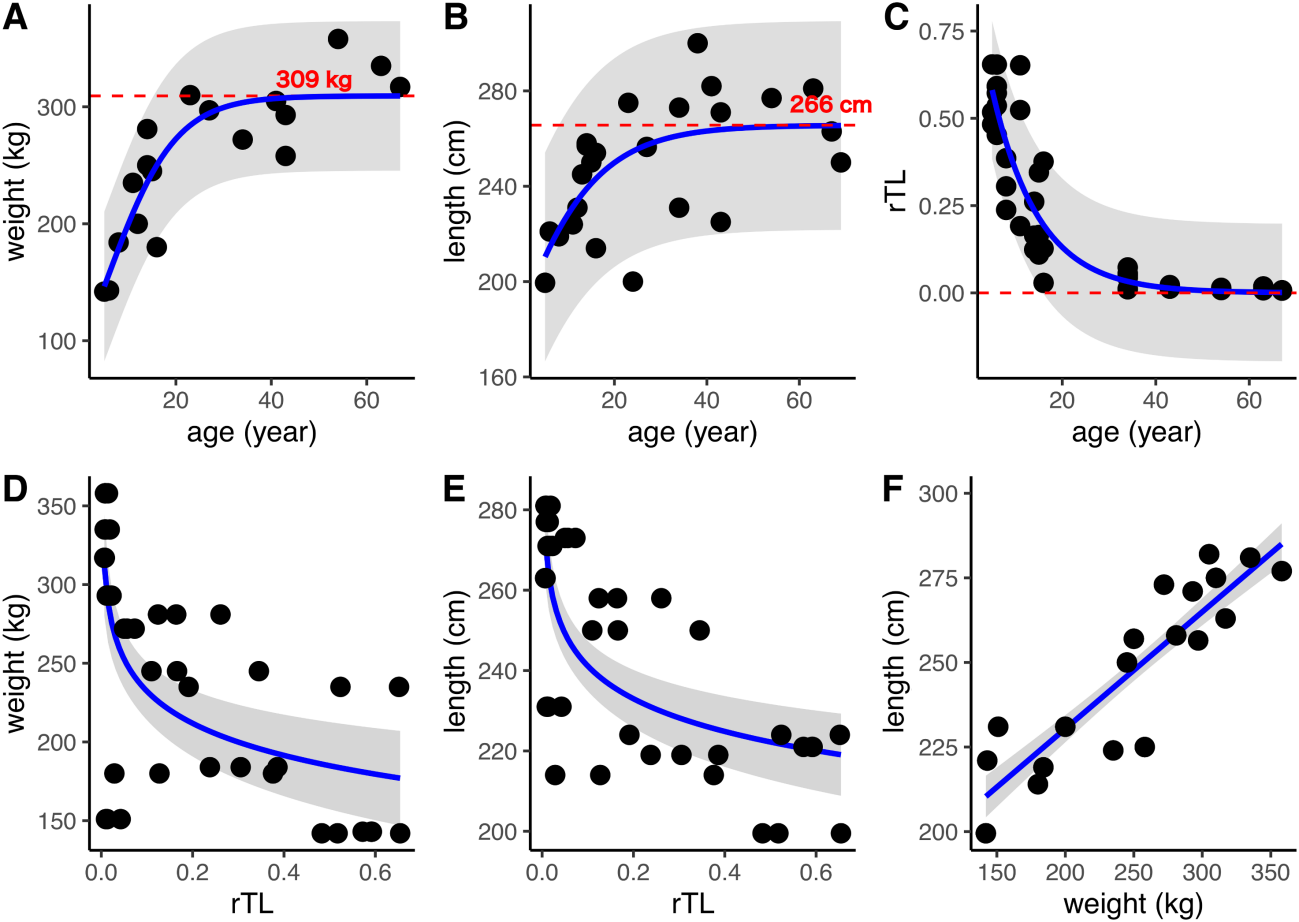
Relationships of age estimation by GLG counts and rTL with body morphometrics. (A) Age by GLGs and body weight; (B) age by GLGs and body length; (C) age by GLGs and rTL; (D) body weight and rTL; (E) body length and rTL; and (F) body length and body weight. Red dashed lines represent the limiting value of the logistic model. 95% confidence interval is indicated by gray stripe. Each individual was done in triplicates and all data were plotted in the graphs.

DNA was isolated from 24 dugong skin samples to measure telomere length by real-time PCR; however, there was positive amplification for only 14 specimens (56%). But all 24 dugong tusks were aged based on counts of GLGs. Using logistic regression, positive correlations were found between age estimated by GLG counts and both body weight and length, with limiting values of approximately 309 kg and 266 cm, respectively (Figs. 3A and 3B; Table 3). By contrast, the relationship between age by GLG counts and rTL fit a negative logistic curve, so increasing age was associated with decreasing telomere lengths (Fig. 3C; Table 3). Negative correlations between rTL and body weight and length were observed, with an adjusted *R*^2^ of 0.43 (*p* < 0.0001) and 0.51 (*p* < 0.0001), respectively, for the model logarithm function (Figs. 3D and 3E; Table 3). Body weight and length were positively correlated with an adjusted *R*^2^ of 0.78 (Fig. 3F; Table 3). In addition, we evaluated the performance of the model obtained from age determination by rTL using a linear regression to test the correlation between the predicted age from rTL and age from GLG counts, resulting in an *R*^2^ of 0.86 and residual standard error of 6.33 (Fig. S1).

**Table 3:** The models fitted to relationships among body weight, body length, rTL and age estimated by GLG counts.

Relationship (*y*~*x*)	Model used	β	*c*	*L*^e^	*R*^2^	AIC	Residual standard error
Weight~age	Positive logistic model^a^	0.14	−0.81	309.33	0.77	181.24	32.61
Length~age	MPositive logistic model^a^	0.09	0.86	265.62	0.39	221.98	22.33
rTL~age	Negative logistic model^b^	−0.10	−3.57	−33.17	0.82	−68.94	0.1005
Weight~rTL	Logarithm model^c^	164.56	−29.39	–	0.43*	427.88	55.48
Length~rTL	Logarithm model^c^	214.08	−11.75	–	0.51*	343.85	18.89
Weight~length	Linear regression model^d^	161.33	0.34	–	0.78*	448.32	11.93

**Notes:**

a $Y = \; \displaystyle{L \over {1 + {e^{ - \left( {c + {\rm \beta} X} \right)}}}}$.

b *Y* $= \; \displaystyle{L \over {1 - {e^{ - \left( {c + {\rm \beta} X} \right)}}}}$.

c $Y = {\rm \beta} \log \left( X \right) + c$.

d $Y = {\rm \beta} X + c$.

e *L* represents the limiting value for logistic function.

* *p*-Value < 0.0001.

### Determining age at maturity

**Figure 4:**
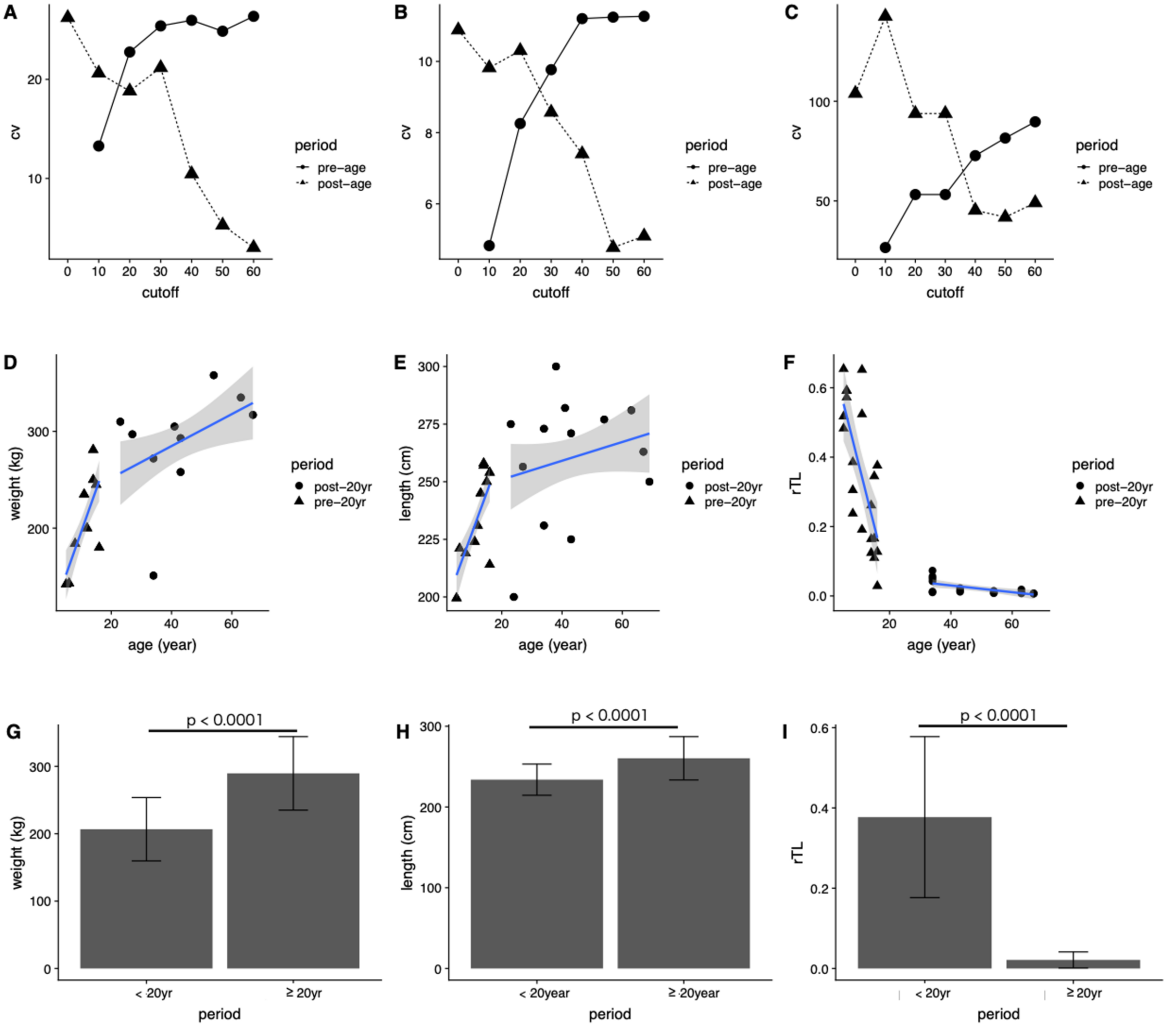
Determining age cutoffs for determining dugong maturity based on body indices and rTL in dugongs. Coefficient of variation (CV) at different age cutoffs every 10 years from 0 to 60 years for (A) weight, (B) length and (C) rTL. Correlations are shown between age and (D) weight, (E) age and length and (F) age and rTL. Average (G) weight, (H) length and (I) rTL are shown for dugongs < or ≥20 years of age.

In general, the CV of dugongs <20 years was higher than that of those ≥20 years in age cutoffs (Figs. 4A and 4B). Differences in body weight and length CVs were noted in dugongs 20–30 years of age (Figs. 4A and 4B). By contrast, the pattern of CV for rTL was the opposite of that for the body indices, although the age cutoff based on a slow rate of telomere attrition was the same; thatis, 20–30 years (Fig. 4C). Taken together, age at physical maturity based on a plateau in growth occurs at an age of 20 years or older.

Based on those results, we used 20 years of age as a cutoff for separating dugongs into physically immature (<20 years) and mature (≥20 years) groups and found significant differences in body weight, body length and rTL between groups (*p* < 0.0001) (Figs. 4G–4I). A higher growth rate was observed for dugongs 5–20 years of age compared to those ≥20 years old (Figs. 4D and 4E). Body weight increased approximately 8.8 kg/year between 5 and 20 years of age, which was reduced to 1.65 kg/year after the age of 20 years (Fig. 4D; Table 4). Body length for the first 20 years increased at a rate of 3.58 cm/year, with no further changes after 20 years (adjusted *R*^2^ = 0.03, *p* = 0.1534). rTL decreased by a value of 0.036/year with an adjusted *R*^2^ = 0.56 (*p* < 0.0001) in immature dugongs, whereas in those ≥20 years of age, it decreased only about 0.001/year (Fig. 4F; Table 4).

**Table 4:** Description statistics of linear regression models for body weight, body length and rTL in dugongs < or ≥20 years of age.

Parameter	Period	β	*c*	Adjusted *R*^2^	*p*-Value
Weight	<20 years	8.80	107.86	0.50	<0.0001
	≥20 years	1.65	218.81	0.15	0.0187
Length	<20 years	3.58	191.60	0.47	<0.0001
	≥20 years	0.41	242.79	0.03	0.1534
rTL	<20 years	−0.036	0.74	0.56	<0.0001
	≥20 years	−0.001	0.07	0.39	0.0032

### Sex differences in growth rates

**Figure 5:**
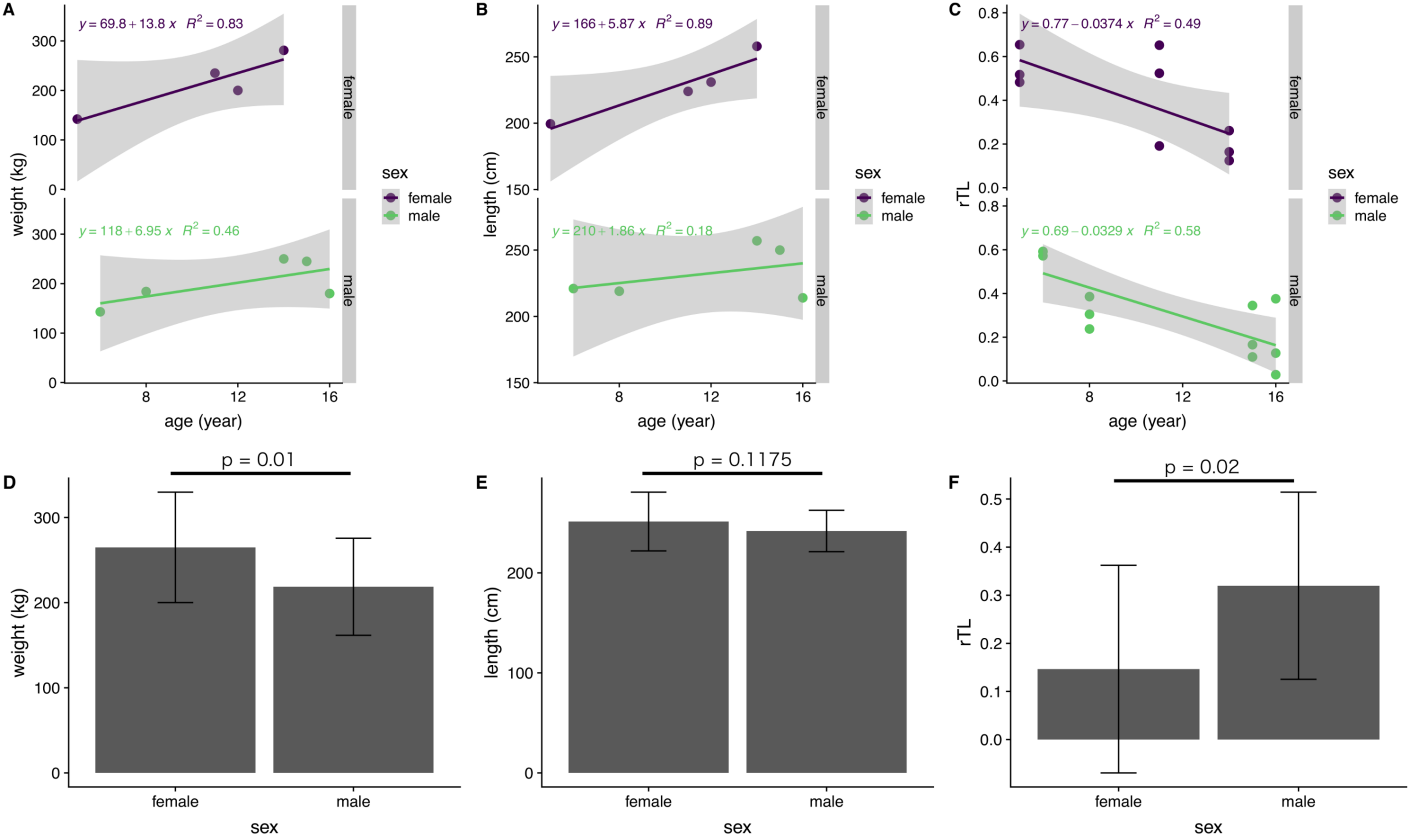
Correlations of age with body indices and rTL, and overall averages for each parameter in female and male immature dugongs related to (A) body weight-age, (B) body length-age, (C) rTL-age, (D) average body weight, (E) average body length and (F) average.

The influence of sex on body indices and rTL was examined for immature dugongs only, because of an absence of data for mature males. There was a significant difference between females and males in body weight, but not body length (Figs. 5D and 5E). Yearly body weight and length increases in immature females (13.8 kg/year and 5.87 cm/year, respectively) were nearly double those observed in males (6.95 kg/year and 2.41 cm/year) (Figs. 5A and 5B). Both genders exhibited a reduction in telomere length of about 0.03 rTL/year, with the average rTL in males being higher than that in females (*p* = 0.02) (Figs. 5C and 5F).

## Discussion

This is the first study to describe the use of telomere length measures for age estimation in dugongs and found a strong correlation between estimated age and telomere shortening. Previously, the only available method to age dugongs relied on counting GLGs in intact tusks of carcasses. The benefits of measuring rTL are the ability to use a variety of tissue types of both living and deceased animals, and that it does not require access to whole tusks. This study found that measurement of rTL via qPCR predicted dugong age using the negative logistic equation “${\rm rTL} = \; \textstyle{{ - 33.17} \over {1 - {e^{\left( {3.57 + 0.1*{\rm age}} \right)}}}}$” and it can be rearranged to ${\rm age} = \; \textstyle{{\ln \left( {1 + \left( {\textstyle{{33.17} \over {\rm rTL}}} \right)} \right) - 3.57} \over {0.1}}$ to determine age of unknown individuals. This model gave a degree of correlation with age estimated by GLG counts with *R*^2^ of 0.86 and an uncertainty for age estimation as approximately ±6.33 years. A strong correlation also was found between body weight and length (*R*^2^ = 0.78) in these dugongs, while modest correlations were found between rTL and body weight (*R*^2^ = 0.43) and length (*R*^2^ = 0.51). Overall, results suggest that rTL is a robust method to estimate age in dugongs, better than body weight and length, and perhaps equal to using counts of tusk GCGs.

The rate of telomere attrition was age-related and more rapid at 0.0036/year in young dugongs aged 5–20 years compared to animals over 20 years of age, when it slowed to 0.001 rTL/year. Studies have shown associations between telomere attrition and age in a wide variety of species, including humans (Daniali et al., 2013) and zebra finches (*Taeniopygia guttata*) (Heidinger et al., 2012). In American alligators (*Alligator mississippiensis*), telomere length was negatively associated with body length as a proxy for age (Scott et al., 2006). In red-sided garter snakes (*Thamnophis sirtalis parietalis*), telomere length decreased with age in males, but not females (Rollings et al., 2017), perhaps as a physiological consequence of sexual dimorphism and energy investment differences in the mating system of this species. Typically, telomere length is measured in peripheral blood leucocytes; however, age-related changes have been documented in over a dozen tissue types, both in mice and humans (Scott et al., 2006). Differences in rTL variability in humans (Dlouha et al., 2014) and rates of stress-induced accelerated aging in mice (Baek et al., 2019) have been shown to be tissue-specific. In contrast, shortening of telomeres in human muscle, skin, fat and leukocytes occurred at equivalent rates, perhaps because stem cells replicate at similar rates among those tissues (Daniali et al., 2013). In the current study, we measured telomere length in skin cells, as these have been used to assess telomere attrition in other species, and shown to correlate with findings in peripheral leukocytes (Dlouha et al., 2014).

Long-lived animals generally exhibit a slower rate of telomere attrition compared to short-lived animals (Whittemore et al., 2019). For example, human (lifespan ~70 years) telomeres shorten at a rate of ~70 bp/year (Canela et al., 2007), while telomeres in mice (lifespan ~5 years) shorten at a rate of 7,000 bp/year (Vera et al., 2012). In other long-lived mammals, telomere shortening occurs at rates of ~110 bp/year in elephants (lifespan ~60 years) (Whittemore et al., 2019), ~770 bp/year in bottlenose dolphins (lifespan ~30–40 years) (Whittemore et al., 2019), ~110 bp/year in American flamingos (*Phoenicopterus ruber*) (lifespan ~40–60 years) (Whittemore et al., 2019) and ~210 bp/year in the griffon vulture (*Gyps fulvus*) (lifespan ~40 years) (Whittemore et al., 2019). Our study showed a high correlation between age and rTL, but it was not possible to calculate numbers of bp reduced per year because we relied on qPCR, a technique that determines a relative change between telomeres and internal gene control (Cawthon, 2002; Olsen et al., 2014), while other techniques like southern blot (Lincz et al., 2020) and quantitative fluorescence in situ hybridization (Canela et al., 2007; Whittemore et al., 2019) calculate actual numbers of base pairs. Still, our study corroborated a relationship between age and telomere shortening in the dugong, similar to other species.

There were significant correlations between age and both body weight and length, explained by positive sigmoid curves. In the first 20 years of life, the relationship between body weight and length was linear (*R*^2^ = 0.50 and 0.47, respectively) and then plateaued, suggesting the dugong is physically mature at about 20 years of age. In immature dugongs (<20 years), body weight increased approximately 8.8 kg/per, and then slowed to 1.65 kg/year in mature animals (≥20 years). Stronger relationships between body morphometrics and age were observed in females compared to males. In addition, in early life, females grew at rates nearly double those of males: 13.8 kg/year and 5.87 cm/year in females and 6.95 kg/year and 2.41 cm/year in males for body weight and length, respectively. A previous study in one pair of captive dugongs reported weight gains of about 42–45 kg/year during the first 10 years of life (Goto et al., 2004), which is higher than what we observed. Possible reasons could be that our study evaluated stranded carcasses, whereas that study was in captive dugongs. Confounding factors affecting growth rates of captive and wild animal can include differences in environmental quality (water, parasites, disease) and food sources. Variations in seagrass nutritional quality are evident (Fortes et al., 2018; Goto et al., 2004; Green & Short, 2003) and may differ from what is fed in captivity. Food consumption in captive dugongs has been reported to be 10–15 kg/day in a calf up to 23–26 kg/day in an adult (Goto et al., 2004). In nature, wild dugongs may not have access to as much seagrass or quantities of food available to captive dugongs. Density of dugongs in the habitat also may affect growth rates due to influences on food availability and habitat quality (Marsh, 1980; Nganvongpanit et al., 2017a, 2017b), which in turn could affect age of sexual maturity. Population differences in age at sexual maturity have been observed in wild dugongs. For example, those from Townsville, northern Queensland, Australia, mature at 10 years of age, compared to 18 years of age along the Papua New Guinean coast (Marsh, 1995). In dugongs along the Andaman sea and Gulf of Thailand coasts, the average mature body length and weight of males and females were 258 and 255 cm, and 250 and 251 kg, respectively (Adulyanukosol, Cherdsukjai & Boukaew, 2011). This was similar to maximum weights of 298 and 293 kg reported in captive male and female dugongs, respectively (Goto et al., 2004). Average lengths of 240–260 cm in adults (Burgess, Lanyon & Keeley, 2012; Marsh, 1980) fit with data based on our equation, where limiting values for body weight and length of dugongs in our study were approximately 309 kg and 266 cm, respectively. In the present study, the absolute age of each animal was unknown, so the standard practice of tusk GLGs counts was used for age estimation (Marsh, 1980; Nishiwaki & Marsh, 1985; Read, Hohn & Lockyer, 2018). Then that chronological age was correlated with body morphometric indices, finding significant relationships with both body weight and length.

There are some limitations to using GLG counts to estimate age in dugongs. One problem is related to poor tusk quality. Tusks are covered by the muzzle until puberty. In females, tusks are rarely observed past the muzzle, whereas in adult males they protrude past the muzzle after the eruption and then can become worn, often resembling the tip of a chisel. Thus, using the tips of tusks below the muzzle, GLG counts may not reveal a true chronological age (Adulyanukosol, Amorena & Miyazaki, 1998; Marsh, 1980). Quantifying GLGs also can be difficult because a clear separation between light and dark bands is not always evident, thus often requiring additional processing steps such as hematoxylin staining (Marsh, 1980). Moreover, dentin layer deposition can be affected by environmental factors, and so rates may vary from year to year (Adulyanukosol, Amorena & Miyazaki, 1998; Marsh, 1980; Nishiwaki & Marsh, 1985). For TL, the source of genomic DNA is a factor and telomeres can shorten at different rates depending on tissue type. For example, bone marrow, endothelium, iliac artery, kidney, lymphocyte, muscle cells, thyroid and dental pulp telomeres shorten at rates of 9, 47–147, 102, 14–60, 41, 24, 90 and 72 bp/year, respectively (Bekaert, De Meyer & Van Oostveldt, 2005; Takubo et al., 2010), while in other tissues (epidermis, bone, cartilage) telomeres do not shorten in relation to aging (Kaewkool et al., 2020). Thus, additional studies in dugongs are warranted to determine actual rates of telomere attrition across different tissue types.

## Conclusion

The dugong is a long-lived marine mammal with a low reproductive rate, and populations are threatened throughout their natural range (Adulyanukosol, Ellen & Potchana, 2010; Burgess, Lanyon & Keeley, 2012; Marsh, 1995). In particular, the incidence of stranding and deaths has dramatically increased, with deleterious effects on dugong population sustainability around the world (Marsh & Sobtzick, 2019; Sahri, Mustika & Murk, 2020). Being able to determine the age of individual dugongs, living or carcass, is important to understanding basic species biology, and also how factors in the environment affect survival and age-related changes in health, reproduction and welfare. We provide strong evidence that rTL can be used for estimating chronological age in this species and have established that physical maturity is reached at about 20 years of age. Thus, our findings offer an alternative approach for assessing age in dugongs that can be conducted using other tissue types and is not reliant on intact tusks, as is the case for GLG counts. This added flexibility makes this technique an important tool to aid in dugong conservation.

## Supplemental Information

10.7717/peerj.10319/supp-1 Supplemental Information 1 The relationship between age estimation of dugong using GLG and rTL under linear regression model.

10.7717/peerj.10319/supp-2 Supplemental Information 2 Dugong raw data.

## Additional Information and Declarations

### Data Availability

The following information was supplied regarding data availability: The raw measurements are available as a Supplemental File.
