# Supplementary figures and images for: Age relationships with telomere length, body weight and body length in wild dugong (Dugong dugon)
Source: PeerJ. 2020 Nov 11;8:e10319. doi: 10.7717/peerj.10319 (PMC7666544; doi:10.7717/peerj.10319)

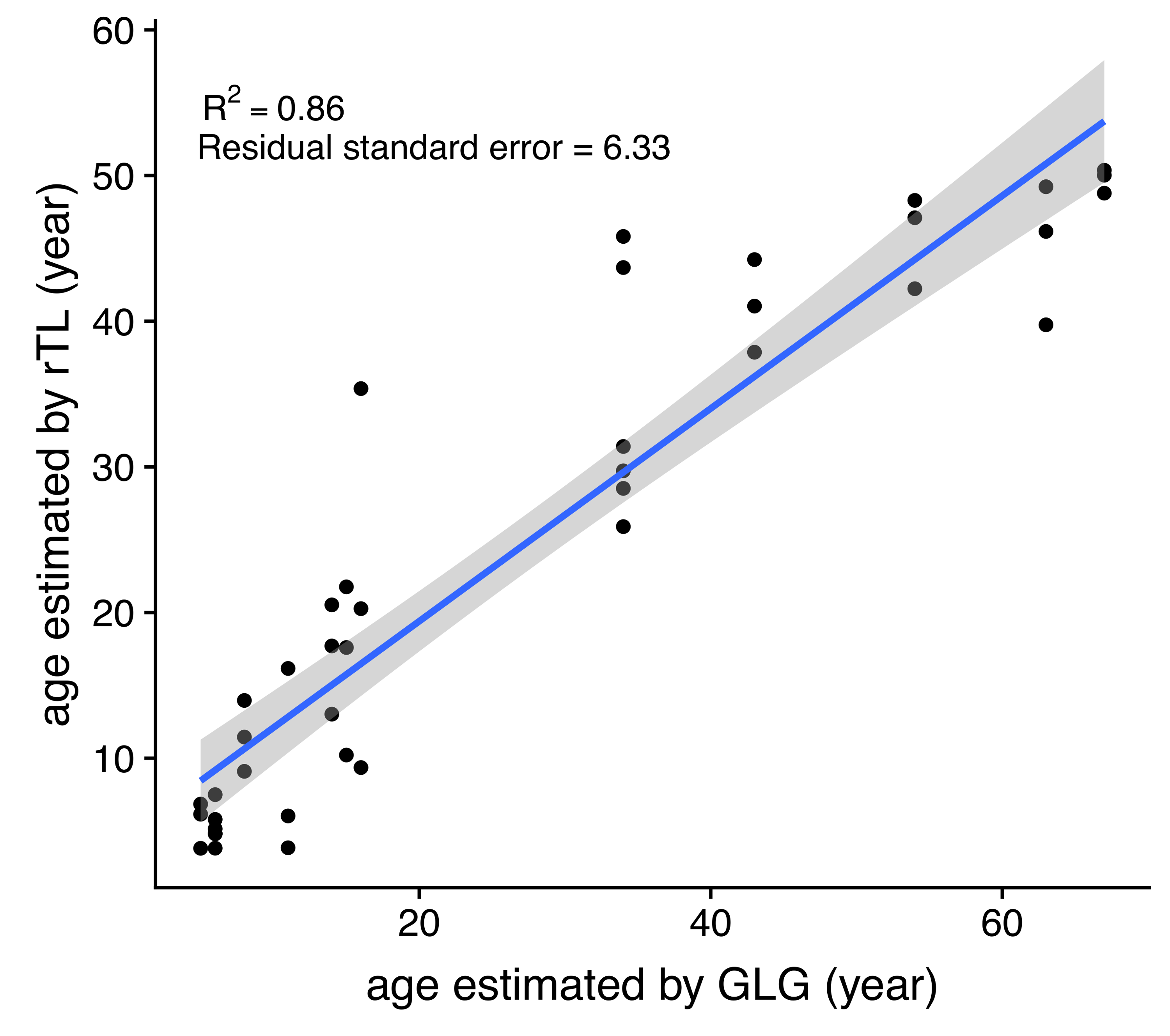

Supplement: Supplemental Information 1 [file peerj-08-10319-s001.png]
